# Supplementary material for: Learning to play against any mixture of opponents
Source: Front Artif Intell. 2023 Jul 20;6:804682. doi: 10.3389/frai.2023.804682 (PMC10400709; doi:10.3389/frai.2023.804682)
Supplement: Supplementary file 1 [file Data_Sheet_1.PDF]

Supplementary Material

1 EXPERIMENTAL DETAILS

Double DQN was used for all experiments van Hasselt et al. (2016). Determining the correct hyperparameters to utilize is a non-trivial problem, because the learning dynamics may vary given different opponent policies. To this end, we sought to find a method for selecting hyperparameters that performs well against a diversity in opponents, while also being computationally tractable to run. We choose hyperparameters that performed best against a uniform-mixture of a fixed set of opponents (five for the soccer environment, and three for cyber-security environment). The opponent policies were generated through PSRO, and were sampled from the resulting strategy sets. For both experiments we also included a random opponent to each strategy set, because we expect this opponent to be one of the more challenging opponents to learn against.

However, there's a chicken-and-egg problem present. In order to utilize PSRO we would also need the aformentioned hyperparameters. As a stop-gap, we choose initial hyperparameters, by evaluating their performance against a random opponent. In summary, for both environments we select hyperparameters by:

1. Sample 200 possible hyperparameter settings, and choose the one that best-performs against a random opponent.
2. Run PSRO until it exceeds a three day walltime.
3. Sample a fixed set of policies from the strategy set generated from PSRO.
4. Sample 200 hyperparameter settings, and evaluate them against uniform mixed-strategy of the four PSRO policies and the random opponent.

We chose to evaluate our hyperparameters against the mixed-strategy opponent, because we believed it offered the most benefit to the baseline method. Future work could look at the interplay of the hyperparameter selection method and the respective performance of both Q-Mixing, and learning a BR directly against a mixed-strategy.

The non-standard hyperparameters listed throughout the appendix are defined as follows:

- **Timesteps** Total number of experiences collected during training.
- **Exploration Fraction** Fraction of the training timesteps used for exploration. The exploration policy is ϵ-greedy, and starts with $\epsilon = 1.0$, and linearly decays to *Exploration Final* hyperparameter.
- **Exploration Final** ϵ The final ϵ value.
- **Training Frequency** Timestep frequency for performing updates.
- **Training Starts** Number of timesteps experienced before training begins.
- **Number of Simulations** The number of simulated episodes performed for evaluation.

1.1 Soccer

The soccer environment is a gridworld comprised of two players, one ball, and two goals. The soccer field is a 5×4 matrix where the goals are off-field on the left and right sides of the field. The ball can spawn in one two positions in the middle of the field, and the players spawn on either side of the spawn points. A graphical representation of the soccer environment can be see in Figure S1. The players are rewarded for moving the ball into the opponent's goal (the one they spawned furthest from).

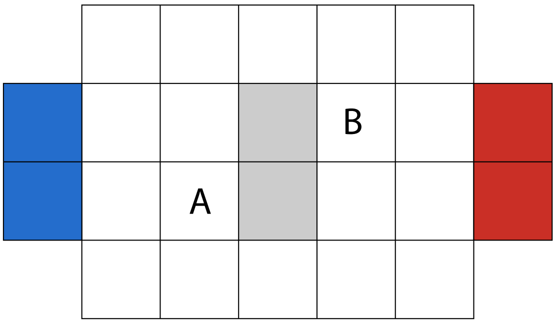

Figure S1. Grid-world soccer environment. The letters represent the respective players. The ball may spawn in either middle highlighted tile, and the player's goal is to score in the opposite net.

In this experiment we select five opponent policies, and hold them fixed throughout the experiments. We consider the hyperparameters in Table S2 as candidates, and found the hyperparameters listed in Table S1 to perform best.

To ensure that each method receives the same simulation budget, we allow each pure-strategy BR 60000 timesteps. An interesting future direction is investigating the trade-off of simulation budget and performance that exists between these methods.

Table S1. Soccer environment hyperparameters against a mixed strategy.

Hyperparameter	Value
Optimizer	Adam
Learning Rate	0.0003
Buffer Size	3000
Gamma	0.99
Timesteps	300000
Batch Size	64
Exploration Fraction	0.33
Exploration Final ϵ	0.01
Training Frequency	1
Training Starts	300

The trained DQN was approximated using a 2 hidden layer neural network. The hidden layers each had 50 units and were fully connected with a ReLU activation. The possible actions are moving in any of the four cardinal directions, or staying in place. The input is a vector of length 120, to represent each of the 20 positions on the board having one of the following states:

- Player 0 is on this square and does not have the ball.

Table S2. Soccer environment considered hyperparameters.

Hyperparameter	Value
Batch Size	32, 64
Buffer Size	300, 1000, 3000, 10000
Learning Rate	1e-3, 3e-3, 1e-4, 3e-4
Timesteps	10000, 30000, 100000, 300000
Exploration Fraction	0.1, 0.3, 0.4, 0.7
Training Starts	100, 300, 1000

- Player 0 is on this square and is holding the ball.
- Player 1 is on this square and does not have the ball.
- Player 1 is on this square and is holding the ball.
- The ball is on the ground on this square.
- Unoccupied space.

1.1.1 Opponent Policy Classifier

The hyperparameters selected for training the opponent classifier are listed in Table S3. The replay buffers gathered from training best-responses against each opponent were merged into one dataset. The classifier was trained to predict the opponent for each observation in the dataset. This resulted in 15000 data points, which were randomly split 90-10 between training and validation.

Table S3. Markov-Soccer opponent policy classifier hyperparameters.

Hyperparameter	Value
Optimizer	Adam
Learning Rate	$5 \cdot 10^{-5}$
Loss	Cross Entropy
Batch Size	64

The classifier was a neural network with the same architecture as a single policy; however, the last layer is modified to choose opponents rather than actions. We did not perform a hyperparameter search on this network or learning algorithm.

1.2 Gathering

The Gathering environment is a gridworld tragedy-of-the-commons game. For motivation and background on the environment please refer to Perolat et al. (2017); Leibo et al. (2017). The observation space in the Gathering environment is the rectangular area in front of the agent stretching 20 cells forward with a width of 10. The agents simultaneous take actions of either moving in the four cardinal directions, rotating left or right, tagging the other agent with a time-out beam, or taking no action. A visual depiction of the environment is provided in Figure S2.

The hyperparameters that were searched over are provided in Table S5 and the final hyperparameters are in Table S4.

1.3 Policy Distillation

In the policy distillation framework, a larger neural network referred to as the "teacher" is used as a training signal for a smaller neural network called the "student". In our experiment the Q-Mixing policy

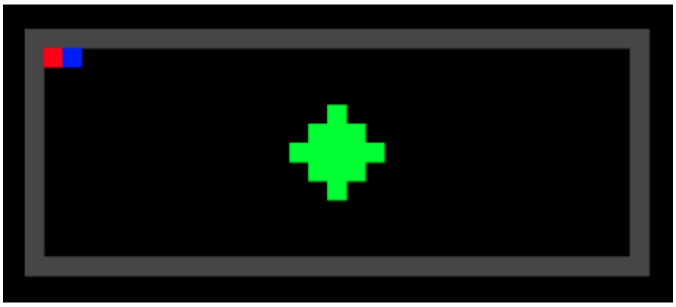

Figure S2. Gathering game map. Players spawn in either the blue or red tile, and apples on the green tiles.

Table S4. Gathering environment hyperparameters.

Hyperparameter	Value
Optimizer	Adam
Learning Rate	$3 \cdot 10^{-4}$
Gradient Norm Clip	None
Buffer Size	30000
Gamma	0.99
Batch Size	64
Exploration Fraction	0.3
Exploration Final ϵ	0.03
Training Starts	1000

Table S5. Gathering environment considered hyperparameters.

Hyperparameter	Value
Learning Rate	3e-3, 1e-4, 3e-4, 1e-5, 3e-5
Gradient Norm Clip	None, 0.1, 1, 10
Buffer Size	3e4, 5e4, 8e5, 1e5
Batch Size	32, 64
Timesteps	3e5, 5e5, 7e5, 1e6, 1.2e6, 1.5e6, 1.7e6, 2e6
Exploration Timesteps	1e5, 2e5, 3e5, 4e5, 5e5, 7e5

is the teacher to a student neural network that is the size of a single BR policy. The student is trained via supervised learning, reusing the pure-strategy BRs' replay buffers as a dataset. A batch of data is sampled from the replay-buffer and the student predicts Q^S the teacher's response Q^T. The student is trained to imitate the softmax policy of the teacher. The full policy distillation loss is

$$\mathcal{L}_{\text{Distill}} = \sum_{i}^{|D|} \text{softmax}(\frac{Q^T}{\tau}) \ln \frac{\text{softmax}(\frac{Q^T}{\tau})}{\text{softmax}(\frac{Q^S}{\tau})},$$

where D is the dataset of concatenated replay buffers.

The hyperparameters used in policy distillation are listed in Table S6. The student policy is the same neural network that's used in computing the best-responses to individual policies; it is described in Section 4.2. We did not perform a hyperparameter search on this network or learning algorithm.

Table S6. Policy distillation hyperparameters.

Hyperparameter	Value
Optimizer	Adam
Learning Rate	0.003
Batch Size	64

REFERENCES

Leibo, J. Z., Zambaldi, V., Lanctot, M., Marecki, J., and Graepel, T. (2017). Multi-agent reinforcement learning in sequential social dilemmas. In *16th International Conference on Autonomous Agents and Multiagent Systems*

Perolat, J., Z. Leibo, J., Zambaldi, V., Beattle, C., Tuyls, K., and Graepel, T. (2017). A multi-agent reinforcement learning model of common-pool resource appropriation. In *31st Conference on Neural Information Processing Systems*

van Hasselt, H., Guez, A., and Silver, D. (2016). Deep reinforcement learning with double Q-learning. In *30th AAAI Conference on Artificial Intelligence*. AAAI, 2094–2100
